# An Autophagy-Related Gene Signature can Better Predict Prognosis and Resistance in Diffuse Large B-Cell Lymphoma

**DOI:** 10.3389/fgene.2022.862179

**Published:** 2022-06-30

**Authors:** Xuan Zhou, Ying-Zhi He, Dan Liu, Chao-Ran Lin, Dan Liang, Rui Huang, Liang Wang

**Affiliations:** ^1^ Second Clinical Medical College of Southern Medical University, Zhujiang Hospital of Southern Medical University, Guangzhou, China; ^2^ Department of Endocrinology, The First Affiliated Hospital of Fujian Medical University, Fuzhou, China; ^3^ Department of Hematology, Zhujiang Hospital of Southern Medical University, Guangzhou, China; ^4^ The First School of Clinical Medicine, Guangdong Medical University, Zhanjiang, China; ^5^ Department of Hematology, Beijing TongRen Hospital, Capital Medical University, Beijing, China

**Keywords:** diffuse large B-cell lymphoma, autophagy-related genes, gene signature, prognosis, autophagy inhibitors

## Abstract

**Background:** Diffuse large B-cell lymphoma (DLBCL) is a highly heterogeneous disease, and about 30%–40% of patients will develop relapsed/refractory DLBCL. In this study, we aimed to develop a gene signature to predict survival outcomes of DLBCL patients based on the autophagy-related genes (ARGs).

**Methods:** We sequentially used the univariate, least absolute shrinkage and selector operation (LASSO), and multivariate Cox regression analyses to build a gene signature. The Kaplan–Meier curve and the area under the receiver operating characteristic curve (AUC) were performed to estimate the prognostic capability of the gene signature. GSEA analysis, ESTIMATE and ssGSEA algorithms, and one-class logistic regression were performed to analyze differences in pathways, immune response, and tumor stemness between the high- and low-risk groups.

**Results:** Both in the training cohort and validation cohorts, high-risk patients had inferior overall survival compared with low-risk patients. The nomogram consisted of the autophagy-related gene signature, and clinical factors had better discrimination of survival outcomes, and it also had a favorable consistency between the predicted and actual survival. GSEA analysis found that patients in the high-risk group were associated with the activation of doxorubicin resistance, NF-κB, cell cycle, and DNA replication pathways. The results of ESTIMATE, ssGSEA, and mRNAsi showed that the high-risk group exhibited lower immune cell infiltration and immune activation responses and had higher similarity to cancer stem cells.

**Conclusion:** We proposed a novel and reliable autophagy-related gene signature that was capable of predicting the survival and resistance of patients with DLBCL and could guide individualized treatment in future.

## Introduction

Diffuse large B-cell lymphoma (DLBCL) accounts for 30% of cases of non-Hodgkin lymphoma (NHL), making it the most common subtype of NHL among adults worldwide (Zhou et al., 2017). Evidence from biological and clinical studies has revealed that there is a striking degree of clinical, phenotypic, and molecular heterogeneity in DLBCL (Reddy et al., 2017). With the advent of the rituximab era, the treatment efficacy and survival status of DLBCL patients have been dramatically improved, and about 2/3 of DLBCL patients could achieve long-term survival. However, the majority of the remaining patients died of disease relapse or drug resistance (Coccaro et al., 2020). To date, the prognostic prediction of DLBCL patients mainly relies on the International Prognostic Index (IPI) and National Comprehensive Cancer Network-International Prognostic Index (NCCN-IPI) (Zhou et al., 2014). As there is lack of information about genes, patients with the same IPI score may still have different prognosis, and the IPI score cannot identify patients with a 5-year overall survival rate of less than 50% (Ruppert et al., 2020). In addition to the commonly used IPI score, the cell of origin according to the Hans model can also predict the patient’s prognosis to a certain extent. Most studies have shown that the prognosis of patients with the germinal center B-cell subtype is better than that of the non-germinal center B-cell subtype (Hans et al., 2004). However, some research results are inconsistent with this conclusion, suggesting that the prognostic stratification ability of the Hans model needs to be further verified (Coutinho et al., 2013). With the rapid development of molecular biology in recent years, researchers have been trying to use sequencing and chip technology to stratify risks and optimize chemotherapy strategies for patients with different types of cancer (Tian et al., 2020).

Meanwhile, autophagy is also one of the breakthrough findings in the field of tumors. It is reported that autophagy is involved in several biological functions, such as apoptosis, immune response, maintenance of cancer stem cell, and drug resistance (Cufí et al., 2011; Sun et al., 2011; Jiang et al., 2019). On one hand, autophagy helps tumor cells to sustain cellular growth by degrading and recycling components of damaged or aged organelles (Katheder et al., 2017). On the other hand, autophagy can maintain the normal cell structure and metabolic stability by removing damaged organelles and DNA in the early stage of tumor, thus suppressing tumor development (Hönscheid et al., 2014). Several studies also showed that antitumor drugs can kill tumor cells by inducing autophagy (Akar et al., 2008; Chiu et al., 2011). As the link between autophagy and tumor prognosis was strengthened, increasing studies have demonstrated the implication of autophagy in DLBCL. Zhang et al. (2020) found that miR-449a downregulated the expression of ATG4B by binding to the 3′UTR of its mRNA, which subsequently reduced the autophagy of T-cell lymphoma cells and promoted tumor apoptosis. Li et al. (2019) showed that CUL4B activated the protective autophagy to promote the growth of DLBCL cells through the JNK signaling pathway, and interfering with the expression of CUL4B could inhibit autophagy by regulating the JNK signaling pathway, thereby decreasing cell proliferation. These findings indicated that autophagy was tightly associated with the progression of DLBCL, and the autophagy-related genes (ARGs) could serve as promising therapeutic targets for DLBCL patients.

The aim of the present study was to construct an autophagy-related gene signature to predict the prognosis of DLBCL patients and explore the differences in pathways, immune response, and tumor stemness between high-risk and low-risk patients.

## Materials and Methods

### Selection of Autophagy-Related Genes

To find genes linked to autophagy, we examined the Human Autophagy Database (http://autophagy.lu/index.html) which included 231 genes reported to be involved in the autophagy process (Moussay et al., 2011). In addition, the term “autophagy” was also searched on the GeneCards website (https://www.genecards.org/) to identify genes that are associated with the autophagy activity. In this study, we defined an association score higher than 7 as autophagy-related genes. After eliminating overlapped genes in the two databases, a total of 309 genes were finally selected for our study.

### Patients’ Samples

Raw data and corresponding clinical information of the GSE31312 and GSE10846 datasets were retrieved from the Gene Expression Omnibus (GEO) database. Transcriptome RNA-sequencing and clinical data on 481 DLBCL samples were obtained from the TCGA database (https://cancergenome.nih.gov/). Patients without clinical survival information or with a follow-up time <0 days were removed from the study. Finally, 234 patients from the TCGA**-**NCICCR dataset and 412 and 470 patients from the GSE10846 and GSE31312 datasets were included in our study. Among them, GSE10846 served as the training cohort, and GSE31312 and NCICCR were used as the validation cohorts.

### Data Processing

For GSE10846 and GSE31312 datasets, the robust multi-array average (RMA) algorithm in the “affy” package in Bioconductor was used to perform background correction, quantile normalization, and final summarization of oligonucleotides per transcript using the median polish algorithm for the raw data (Zeng et al., 2019). In addition, the probes were annotated according to the “hgu133plus2.db” package. If multiple probes corresponded to the same gene, the largest average value was calculated as the expression value of this gene. Finally, K Nearest Neighbor (KNN) imputation was used to impute missing expression values in the gene expression profiles (Yasrebi, 2015). For the NCICCR dataset, the IDs were annotated based on the human genome reference (Hg38). In the event of multiple IDs matching to the same gene, the genes with the largest average value were served. Then, the voom algorithm from the “limma” package was used for data normalization (Ducie et al., 2017).

### Construction of a Gene Signature Associated With Survival of Diffuse Large B-Cell Lymphoma Patients

Univariate Cox proportional hazard regression analysis was first conducted to screen the genes associated with overall survival (OS). Genes with *p* < 0.05 were considered statistically significant. Then, the intersection of prognosis-related genes and autophagy-related genes was taken to obtain prognostic-related autophagy genes. These genes were further screened by LASSO regression analysis and multivariate Cox regression analysis. We calculated the riskscore for each patient by using the regression coefficients of the individual genes obtained from the multivariate Cox regression model and the expression value of each of the selected genes. The formula was as follows: $\text{Riskscore}=\;\overset{n}{\underset{i=1}{\sum }}\text{expi}*\text{βi}$ . Patients in each cohort were classified into high-risk and low-risk groups by using the median riskscore.

### Development of a Nomogram

A nomogram was constructed based on the results of multivariate analysis. The performance of the nomogram was measured by area under the ROC curve and the calibration curve, and its predictive ability was further verified in the validation cohort. X-tile software was used to find the best cutoff value of the nomogram score, according to the highest χ^2^-value defined by the Kaplan–Meier survival analysis and log-rank test (Camp et al., 2004).

### Functional and Pathway Analysis

The correlation test was used to identify genes correlated with the autophagy-related genes of the gene signature under the cutoff value of the absolute value of the correlation coefficient which was higher than 0.4, and the p value was lower than 0.05. Gene Ontology (GO) and Kyoto Encyclopedia of Genes and Genomes (KEGG) pathway enrichment analyses were then performed with these hooked genes by the “clusterProfiler” package to further explore the function of the autophagy-related genes of the gene signature. In addition, we performed Gene Set Enrichment Analysis (GSEA) to uncover the difference in signaling pathways between high-risk and low-risk groups.

### Estimation of TME Cell Infiltration

The ESTIMATE algorithm was performed to quantify the tumor microenvironment, including the immune score and stroma score. Moreover, we obtained gene sets of each TME infiltrating immune cell type from the study of Charoentong et al. (2017), which included activated dendritic cells, macrophages, activated CD8^+^ T cells, regulatory T Cells, and natural killer T cells. Subsequently, ssGSEA in the “GSVA” package based on the deconvolution algorithm was used to estimate the relative infiltration level of each cell population in each DLBCL sample with gene expression data.

### Correlation Between the Gene Signature and Immune Activation-Related Genes and Immune Activation Pathways

In order to explore a potential relationship between the gene signature and immune response, we first selected TNF, IFNG, TBX2, GZMB, CD8A, PRF1, GZMA, CXCL9, and CXCL10 that were extracted from the published literature and considered to be associated with immune activation. We further downloaded immune activation gene sets from KEGG, which included antigen processing and presentation, NOD-like receptor, T-cell receptor, and Toll-like receptor (Zeng et al., 2019).

### Correlation Between the Gene Signature and Tumor Stemness

To explore the relationship between the gene signature and tumor stemness, we used one-class logistic regression (OCLR) algorithm to calculate the gene expression-based stemness index (mRNAsi) of DLBCL patients, and mRNAsi was then mapped to the range of 0–1, utilizing a linear transformation that was subtracted by the minimum and divided by the maximum (Malta et al., 2018). MRNAsi could describe the similarity between tumor cells and stem cells, and it might be considered a quantitative form of CSCs. Those patients with high mRNAsi scores were associated with active biological processes and a higher level of tumor dedifferentiation.

### Statistical Analysis

All analyses were carried out by R version 3.6.1 and corresponding packages. We applied the Wilcoxon test for continuous variables to compare the differences between high-risk and low-risk groups. The Coxph function in the “survival” package was used for univariate and multivariate Cox regression analyses, and the “glmnet” package was performed for Lasso regression analysis. Kaplan–Meier curves were plotted by the “survival” package, and the log-rank test was used to analyze the significant difference in overall survival and progression-free survival of high-risk and low-risk DLBCL patients. The area under the curve (AUC) of the ROC curve was calculated by the “survival ROC” package to evaluate the accuracy of the gene signature. The “rms” package was used to generate a nomogram. A normalized enrichment (NES) and p-adjusted were used to determine the statistical significance of GSEA analysis.

## Results

### Construction and Validation of the Gene Signature

**FIGURE 1:**
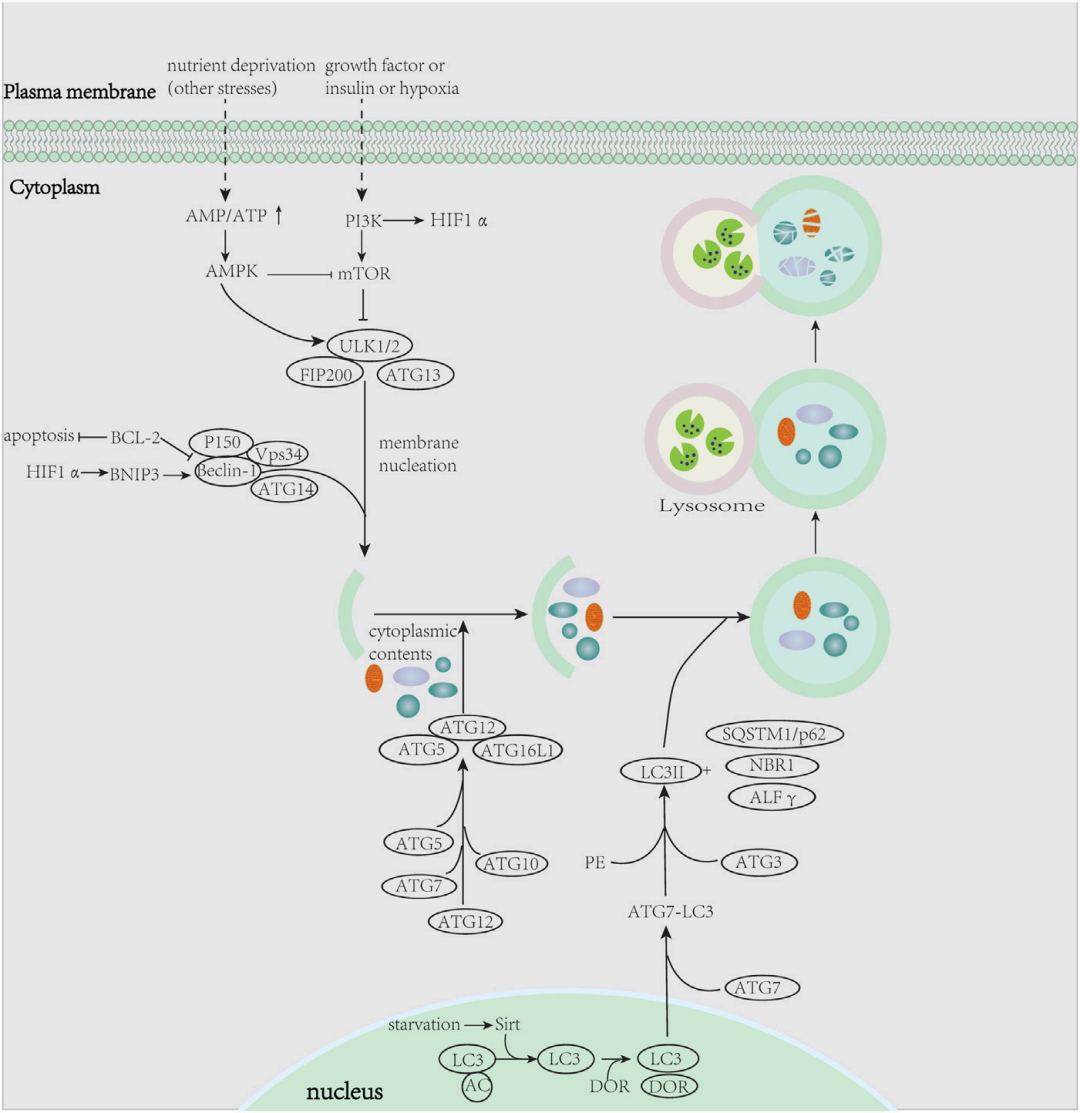
Summary of the process of autophagy.

**FIGURE 2:**
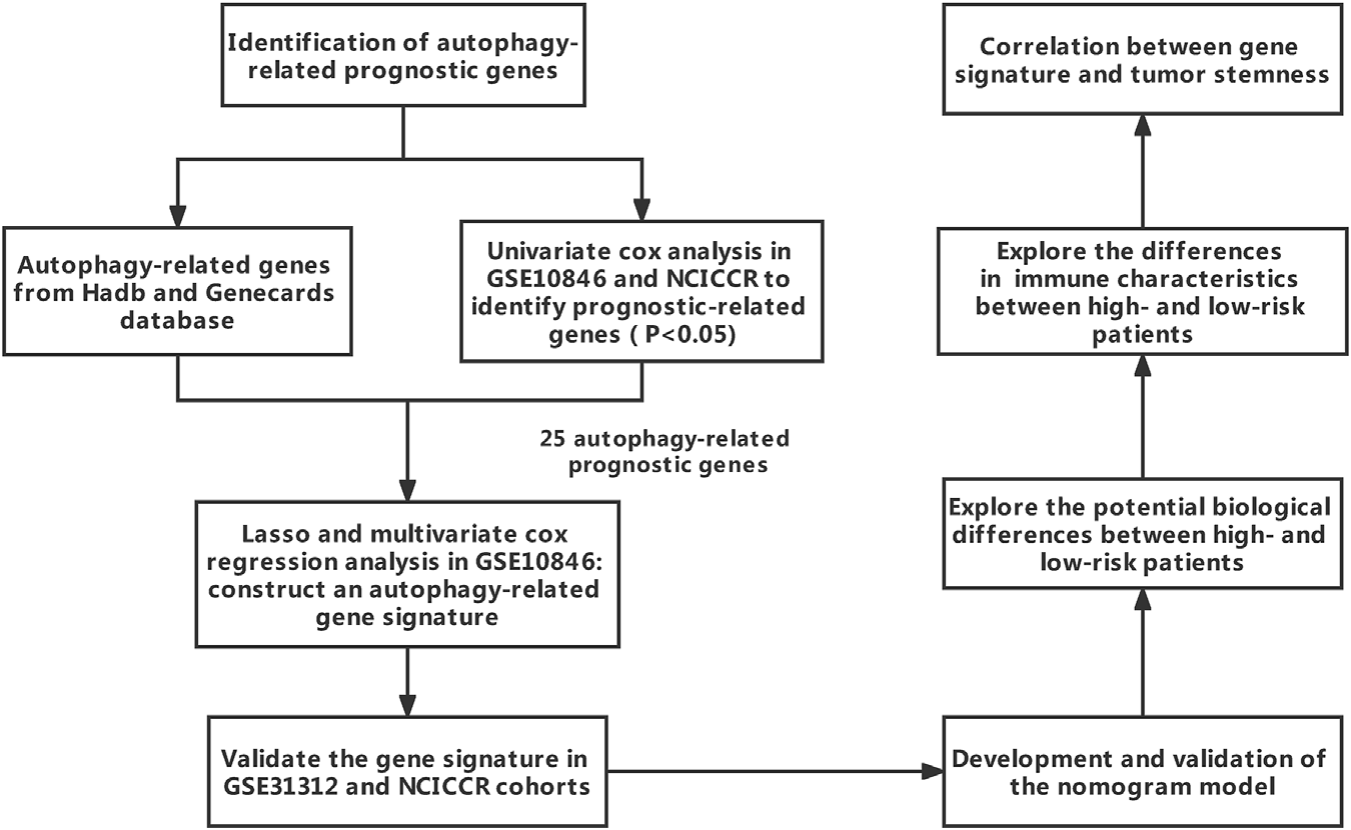
Study flowchart.

**FIGURE 3:**
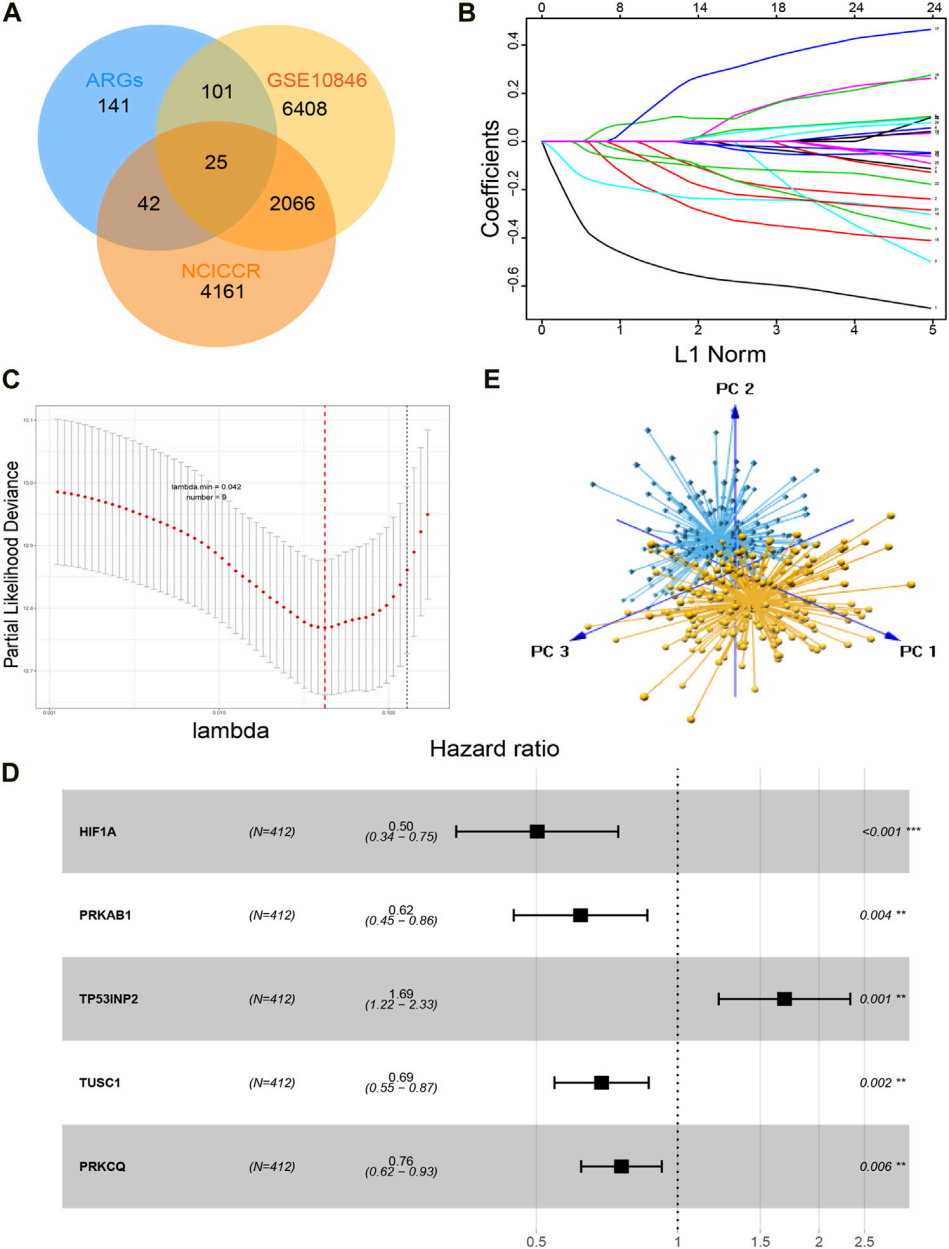
Construction of the gene signature. **(A)** Overlapped genes generated by autophagy-related genes and prognosis-related genes of GSE10846 and NCICCR datasets. **(B)** LASSO coefficient profiles of the 25 overlapped genes. **(C)** Nine genes are selected by LASSO Cox regression analysis. Two dotted vertical lines are drawn at the value of minimum criteria and 1-s.e criteria of cross-validation, respectively. **(D)** Five genes that make up the gene signature and their corresponding hazard ratios resulting from multivariable Cox regression. **(E)** Principal component analysis of the five autophagy-related genes between high-risk and low-risk patients. Low-risk samples are marked in blue, and high-risk samples are marked in yellow.

**FIGURE 4:**
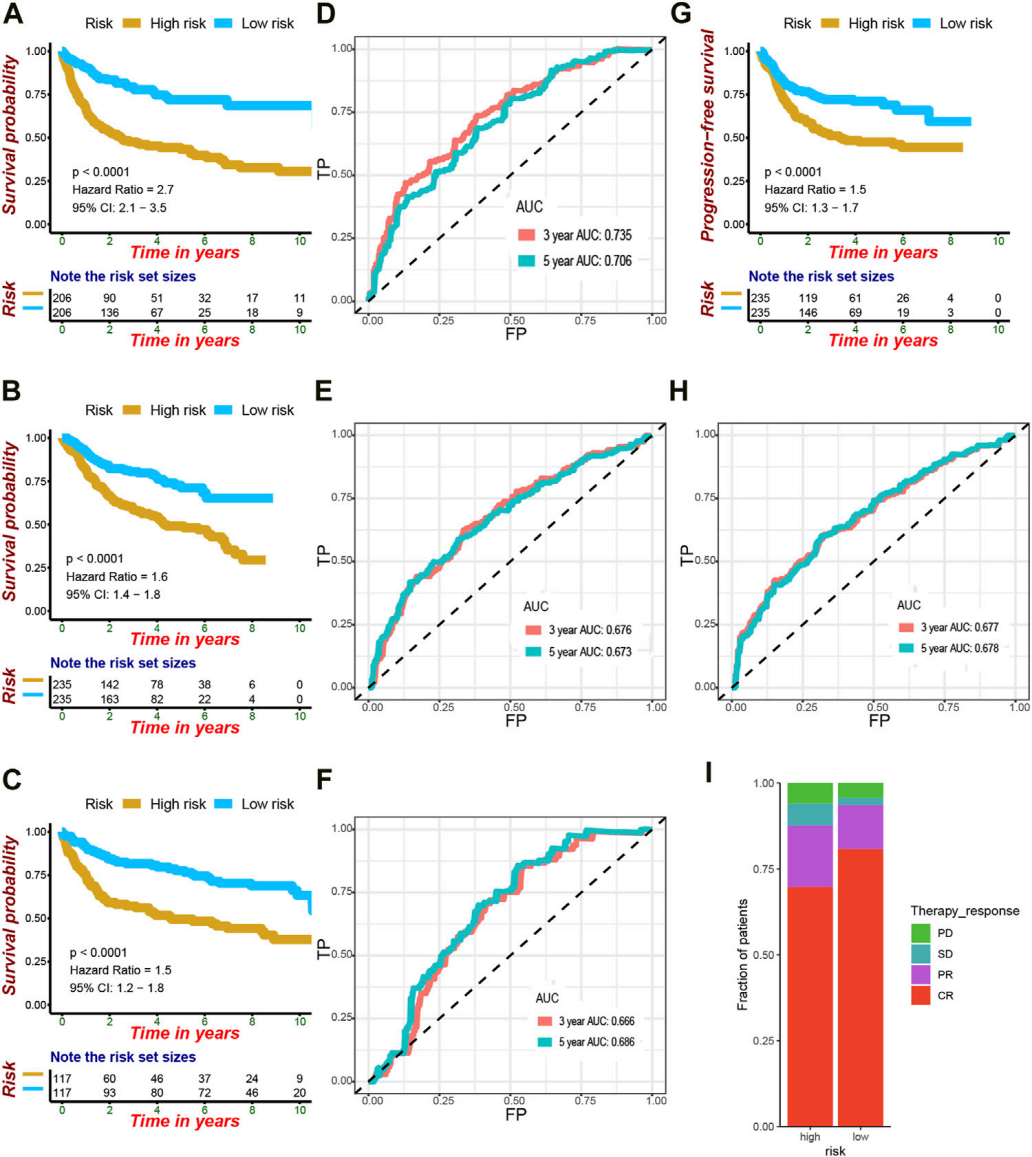
Verification of the efficacy and accuracy of the gene signature. **(A)** Kaplan–Meier analysis of OS for low-risk and high-risk patients in the training cohort. **(B)** Kaplan–Meier analysis of OS for low-risk and high-risk patients in the GSE31312 cohort. **(C)** Kaplan–Meier analysis of OS for low-risk and high-risk patients in the NCICCR cohort. **(D)** Area under the ROC curve at 3 and 5 years to evaluate the accuracy of OS prediction in the training cohort. **(E)** Area under the ROC curve at 3 and 5 years to evaluate the accuracy of OS prediction in the GSE31312 cohort. **(F)** Area under the ROC curve at 3 and 5 years to evaluate the accuracy of OS prediction in the NCICCR cohort. **(G)** Kaplan–Meier curves showing progression-free survival of high-risk and low-risk patients based on the GSE31312 cohort. **(H)** Area under the ROC curve at 3 and 5 years to evaluate the accuracy of PFS prediction in the GSE31312 cohort. **(I)** Difference in the therapy response between high-risk and low-risk patients in the GSE31312 cohort.

Figure 1 summarizes the process of autophagy, the key pathways, and autophagy-related genes involved. Figure 2 showed the study flowchart. The clinical information of DLBCL patients from the GSE31312, GSE10846, and NCICCR datasets is shown in Table 1. In order to identify prognosis-related genes, we respectively performed univariate Cox regression analysis in NCICCR and GSE10846 datasets. Under the cutoff values of *p* < 0.05, 8600 genes in GSE10846 and 6294 genes in the NCICCR dataset were considered as prognosis-related genes. By overlapping the prognosis-related genes with autophagy-related genes, 25 shared genes were retained (Figure 3A). These significant genes were afterward entered into LASSO regression analysis and multivariate Cox regression analysis, and the GSE10846 dataset served as the training cohort (Figures 3B,C). Finally, TP53INP2, PRKCQ, TUSC1, PRKAB1, and HIF1A were identified as members of the gene signature (Figure 3D). TP53INP2 with HR > 1 was regarded as a risk gene, while remaining genes with HR < 1 as protective genes. According to the relative expression level of five genes and the corresponding multivariate Cox regression coefficient, the riskscore of each sample in the training and validation cohorts could be calculated easily. The riskscore was calculated as follows: Riskscore = (0.524 × TP53INP2 expression)—(0.276 × PRKCQ expression)—(0.373 × TUSC1 expression)—(0.476 × PRKAB1 expression)—(0.689 × HIF1A expression). The patients in each cohort were classified into high-risk and low-risk groups according to the median value of the riskscore. Principal component analysis (PCA) based on the five autophagy-related genes confirmed the difference between the two groups (Figure 3E). Kaplan–Meier curves demonstrated that the patients in the high-risk group had shorter overall survival (OS) than those in the low-risk group (HR: 2.7, 95%CI: 2.1–3.5, and *p* < 0.001; Figure 4A). The finding was further validated in external cohorts to evaluate the reproducibility and validity of this gene signature (GSE31312: HR: 1.6, 95% CI: 1.4–1.8, and *p* < 0.001; NCICCR: HR: 1.5, 95% CI: 1.2–1.8, and *p* < 0.001; Figures 4B,C). In predicting the 3-year and 5-year OS rate of DLBCL patients, the gene signature achieved AUC values of 0.735 and 0.706 in the GSE10846 cohort, 0.676 and 0.673 in the GSE31312 cohort, and 0.666 and 0.686 in the NCICCR cohort, respectively, showing a substantially effective performance for overall survival prediction (Figures 4D–F). In addition, shorter progressive-free survival (PFS) was found in the high-risk patients of the GSE31312 cohort (HR: 1.5, 95%CI: 1.3–1.7; Figure 4G). The gene signature also showed good performance in predicting PFS with 3-year AUC and 5-year AUC of 0.677 and 0.678 (Figure 4H). In addition, patients with low-risk in the GSE31312 cohort showed a higher rate of remission rate (Figure 4I).

**TABLE 1:** Summary of DLBCL patients clinical characteristics.

	GSE10846 (*n* = 412)	GSE31312 (*n* = 470)	NCICCR(*n* = 234)
Gender			
Male	222 (53.9%)	271 (57.7%)	139 (59.4%)
Female	172 (41.7%)	199 (42.3%)	95 (40.6%)
Unknown	18 (4.4%)	−	−
Age (years)			
≤60	188 (45.6%)	200 (42.6%)	116 (49.6%)
>60	224 (54.4%)	270 (57.4%)	118 (50.4%)
Ann Arbor Stage			
I/II	188 (45.6%)	220 (46.8%)	109 (46.6%)
III/IV	217 (52.7%)	250 (53.2%)	121 (51.7%)
Unknown	7 (1.7%)	−	4 (1.7%)
ECOG			
<2	295 (71.6%)	374 (79.6%)	−
≥2	93 (22.6%)	96 (20.4%)	−
Unknown	24 (5.8%)	−	−
Subtype			
GCB	182 (44.2%)	248 (52.8%)	−
Non-GCB	230 (55.8%)	222 (47.2%)	−
N_extra			
<2	351 (85.2%)	366 (77.8%)	−
≥2	30 (7.3%)	104 (22.1%)	−
Unknown	31 (7.5%)	−	−
IPI			
≤2	−	274 (58.3%)	126 (53.9%)
>2	−	150 (31.9%)	67 (28.6%)
Unknown	−	46 (9.8%)	41 (17.5%)
Regimen			
RCHOP	232 (56.3%)	470 (100%)	−
CHOP	180 (43.7%)	−	−
Therapy response			
CR	−	354 (75.3%)	−
PR	−	72 (15.3%)	−
SD	−	20 (4.3%)	−
PD	−	24 (5.1%)	−

**FIGURE 5:**
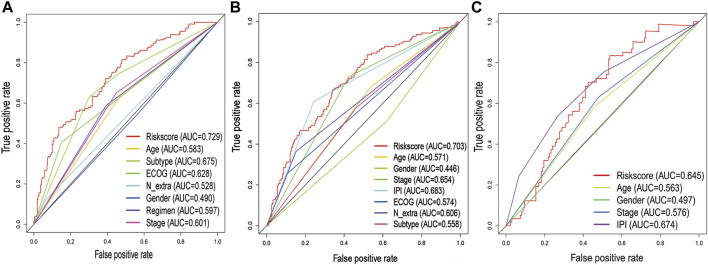
Comparison of 3-year ROC curves with other common clinical characteristics showed the superiority of the gene signature. **(A)** GSE10846 dataset. **(B)** GSE31312 dataset. **(C)** NCICCR dataset.

After removing patients with incomplete clinical information, we analyzed the correlation between gene signature and clinical factors. The results suggested that the gene signature was related to the ECOG score and subtype, but there was no obvious correlation with other clinical factors (Supplementary Figure S1). In addition, the multi-index ROC curve indicated that the predictive accuracy of the gene signature was higher than that of the clinicopathological factors, even higher than the IPI score in the GSE31312 cohort (Figure 5).

### Gene Signature Is Independent of Other Clinicopathological Factors

We further evaluated the prognostic value of the gene signature and other clinicopathological factors by using univariate and multivariate Cox regression analyses. It suggested that the gene signature may serve as a valuable prognostic parameter, independent of other clinical factors (GSE10846: HR: 1.830, 95% CI: 1.218–2.750, *p* = 0.004; NCICCR: HR: 2.444, 95% CI: 1.605–3.723, *p* < 0.001; GSE 31312: HR: 1.984, 95% I: 1.417–2.777, *p* < 0.001; Table 2 and Table 3). When stratified by age, subtype, stage, regimen, ECOG, and IPI score, the gene signature was still a clinically and statistically significant prognostic model (Table 4).

**TABLE 2:** Univariate Cox regression analysis of overall survival in the training and validation cohorts.

Variables	Training cohort		NCICCR cohort		GSE31312 cohort	
	HR (95%CI)	p	HR (95%CI)	p	HR (95%CI)	p
Age						
(>60 vs. ≤60)	2.015 (1.422–2.855)	<0.001	1.963 (1.296–2.972)	0.001	1.850 (1.336–2.560)	<0.001
Gender						
(Men vs. Women)	0.912 (0.654–1.270)	0.585	1.223 (0.804–1.860)	0.348	0.966 (0.712–1.310)	0.824
Subtype						
(Non-GCB vs. GCB)	2.698 (1.858–3.918)	<0.001	−	−	1.512 (1.117–2.047)	0007
Regimen						
(CHOP vs. RCHOP)	1.917 (1.343–2.737)	<0.001	−	−	−	−
ECOG						
(≥2 vs. <2)	2.884 (2.049–4.058)	<0.001	−	−	2.037 (1.460–2.841)	<0.001
Stage						
(III/IV vs. I/II)	1.917 (1.357–2.709)	<0.001	1.500 (0.996–2.260)	0.052	2.337 (1.688–3.238)	<0.001
N_extra						
(≥2 vs. <2)	1.869 (1.089–3.207)	0.023	−	−	2.202 (1.597–3.038)	<0.001
Riskscore						
(High vs. Low)	2.608 (1.824–3.728)	<0.001	2.457 (1.616–3.736)	<0.001	2.326 (1.680–3.220)	<0.001

**TABLE 3:** Multivariate Cox regression analysis of overall survival in the training and validation cohorts.

Variable	Training cohort		NCICCR cohort		GSE31312 cohort	
	HR (95%CI)	*p*	HR (95%CI)	*p*	HR (95%CI)	*p*
Age						
(>60 vs. <=60)	1.801 (1.261–2.573)	0.001	1.945 (1.283–2.949)	0.002	1.496 (1.068–2.096)	0.019
Gender						
(Men vs. women)	−	−	−	−	−	−
Subtype						
(Non-GCB vs. GCB)	1.785 (1.188–2.682)	0.005	−	−	1.361 (0.997–1.856)	0.051
Regimen						
(CHOP vs. RCHOP)	1.757 (1.141–2.705)	0.01	−	−	−	−
ECOG						
(≥2 vs. < 2)	2.138 (1.486–3.076)	<0.001	−	−	1.745 (1.233–2.467)	0.001
Stage						
(III/IV vs. I/II)	1.436 (0.995–2.072)	0.053	−	−	1.725 (1.207–2.463)	0.002
N_extra						
(≥2 vs. < 2)	2.293 (1.227–4.286)	0.009	−	−	1.623 (1.152–2.285)	0.019
Riskscore						
(High vs. low)	1.830 (1.218–2.750)	0.004	2.444 (1.605–3.723)	<0.001	1.984 (1.417–2.777)	<0.001

**TABLE 4:** Stratified analysis for DLBCL patients based on the gene signature.

	Events (n)/patients (N)			HR (95%CI)	*p*-value
	All patients	Low risk	High risk		
Age					
≤60	52/188	13/98	39/90	2.8 (1.8–4.3)	*p* < 0.0001
>60	111/224	35/108	76/116	2.7 (2.0–3.5)	*p* < 0.0001
Subgroup					
GCB	48/182	20/123	28/59	3.9 (2.4–6.4)	*p* < 0.0001
Non_GCB	115/230	28/83	87/147	2.1 (1.5–2.8)	*p* = 0.0002
Stage					
I/II	57/188	18/102	39/86	3.0 (1.9–4.5)	*p* = 0.0264
III/IV	102/217	28/99	74/118	2.6 (1.9–3.4)	*p* < 0.0001
Regimen					
RCHOP	59/232	30/152	29/80	2.6 (1.7–3.9)	*p* = 0.0008
CHOP	104/170	18/54	86/126	2.6 (1.9–3.7)	*p* < 0.0001
ECOG					
<2	97/295	30/151	67/144	2.5 (1.9–3.4)	*p* < 0.0001
≥2	60/93	16/40	44/53	3.1 (2.0–4.8)	*p* < 0.0001
N_extra					
<2	139/351	41/175	98/176	2.9 (2.2–3.8)	*p* < 0.0001
≥2	16/30	5/14	11/16	1.7 (0.8–3.3)	*p* = 0.1272
IPI					
≤2	70/274	16/142	54/132	1.7 (1.4–2.2)	*p* < 0.0001
>2	85/150	26/61	59/89	1.4 (1.2–1.7)	*p* = 0.0146

In addition, we further examined the effect of treatment response on survival. The response to treatment was found to be significantly correlated with the survival rate (Supplementary Figure S2A). The median OS time was not yet reached in patients with complete response, and the median OS time was 1.429, 0.936, and 0.341 years in patients with partial response, stable disease, and progressive disease, respectively. We also found that the gene signature could divide patients achieving complete response into two groups with significant differences in survival (Supplementary Figure S2B). This result indicated that achieving CR did not always mean a favorable prognosis. However, the gene signature did not have the ability to further distinguish the prognosis of patients with partial response, stable disease, and progressive disease (Supplementary Figure S2C–E).

### Personalized Prognostic Prediction Nomogram

**FIGURE 6:**
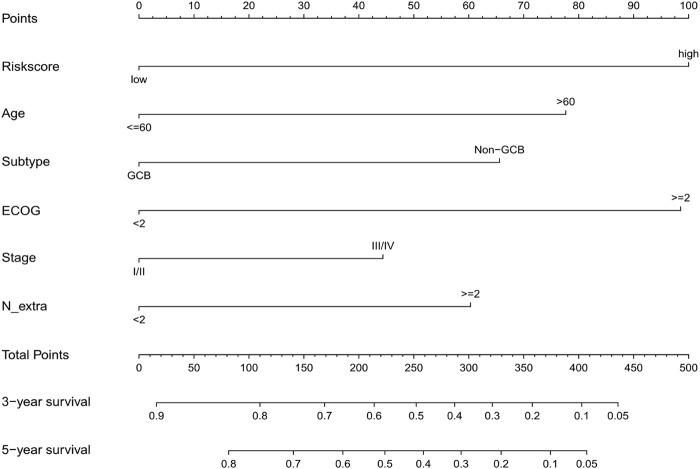
Predictive nomogram was constructed using clinical risk factors and the gene signature.

**FIGURE 7:**
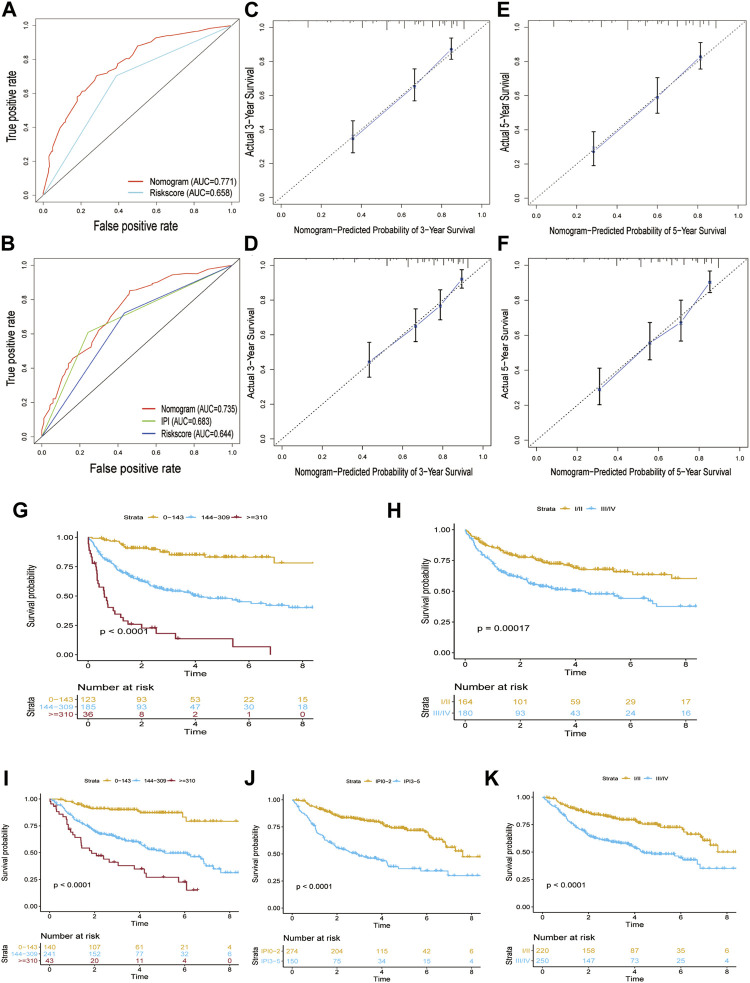
Verification of the performance of the nomogram model. **(A)** Area under the ROC curve at 3 years was used to assess the prognostic accuracy of the nomogram in the GSE10846 dataset. **(B)** Area under the ROC curve at 3 years was used to assess the prognostic accuracy of the nomogram in the GSE31312 dataset. **(C)** Calibration curves of the nomogram in prediction of the 3-year OS in the GSE10846 dataset. **(D)** Calibration curves of the nomogram in prediction of the 3-year OS in the GSE31312 dataset. **(E)** Calibration curves of the nomogram in prediction of the 5-year OS in the GSE10846 dataset. **(F)** Calibration curves of the nomogram in prediction of the 5-year OS in the GSE31312 dataset. **(G)** Kaplan–Meier curves showing the survival difference between different nomogram score groups in the GSE10846 dataset. **(H)** Kaplan–Meier curves showing the survival difference between different stages in the GSE10846 dataset. **(I)** Kaplan–Meier curves showing the survival difference between different nomogram score groups in the GSE31312 dataset. **(J)** Kaplan–Meier curves showing the survival difference between different IPI score groups in the GSE31312 dataset. **(K)** Kaplan–Meier curves showing the survival difference between different stages in the GSE31312 dataset.

We generated a nomogram to predict the probability of 3-year and 5-year OS by integrating the gene signature and clinical factors (Figure 6). In order to better conduct external verification, we converted the gene signature into a binary variable. The AUC at 3 years of the nomogram in the training cohort was 0.771, and the AUC of the gene signature was 0.658 (Figure 7A). The GSE31312 cohort was used to validate the predictive accuracy of the nomogram, and the AUC at 3 years of the nomogram in the GSE31312 cohort was 0.735, which is higher than that of the gene signature and IPI score (Figure 7B). In addition, despite the training cohort or the validation cohorts, the calibration plot had a favorable agreement between the prediction by the nomogram and ideal model in the probability of 3-year and 5-year survival (Figures 7C–F).

We defined the optimal cutoff value of the nomogram score based on the X-tile plots, and patients in both the training and validation cohort were separately stratified into three subgroups according to the cutoff value (0–143, 144–309, and ≥310). In the training cohort, the 5-year OS rate of the nomogram model was 83.1, 47.9, and 13.6%, respectively, while the 5-year OS rate of the Ann arbor stage I–II and III–IV was 67.6 and 47.6%, respectively (Figures 7G,H). In the validation cohort, the 5-year OS rates of the nomogram model were 83.2, 50.7, and 22.5%, respectively, while the 5-year OS rates of the IPI score 0–2 and 3–5 were 72.8 and 36.4%, respectively, and those of Ann arbor stage I–II and III–IV were 74 and 48%, respectively. Therefore, the nomogram displayed better prognostic stratification ability than the IPI score and Ann Arbor staging system (Figures 7I–K).

### Identification of Involved Functions and Signaling Pathways

**FIGURE 8:**
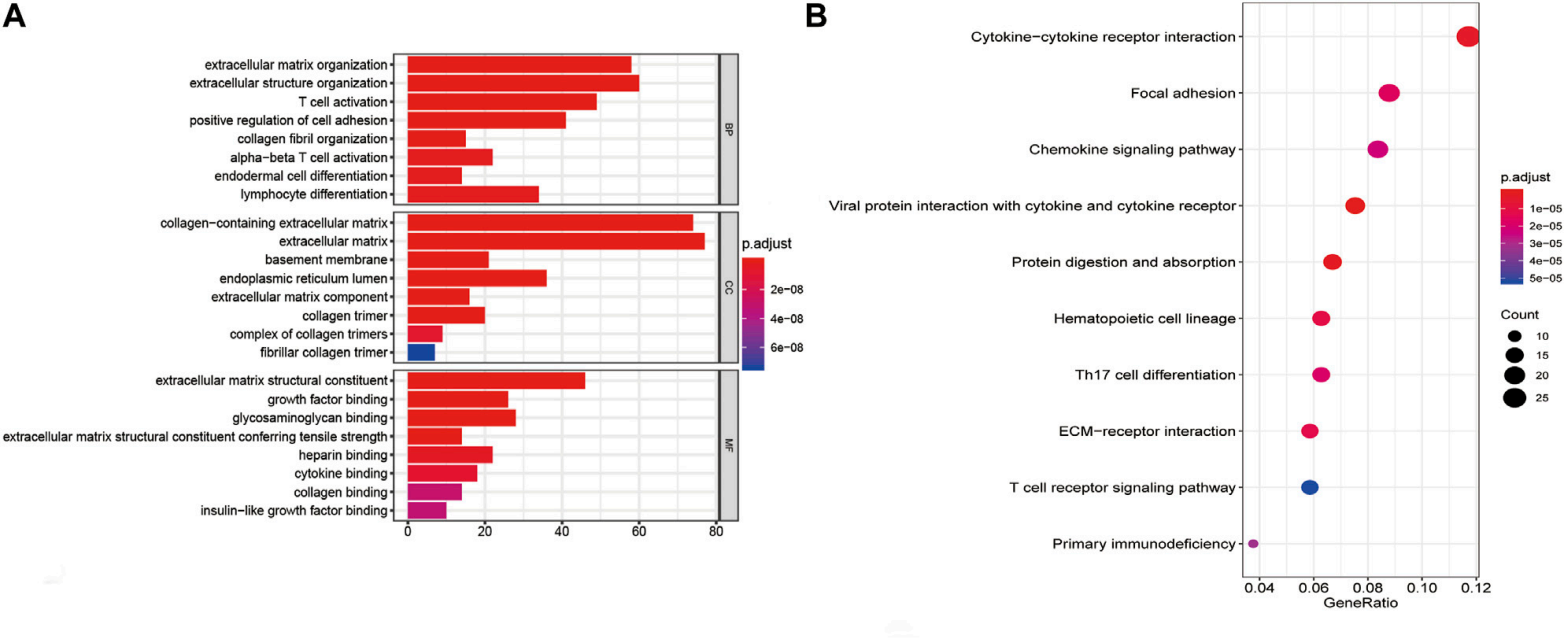
Potential functions and signaling pathways related to the autophagy-related genes of the gene signature. **(A)** GO analysis. **(B)** KEGG analysis.

**FIGURE 9:**
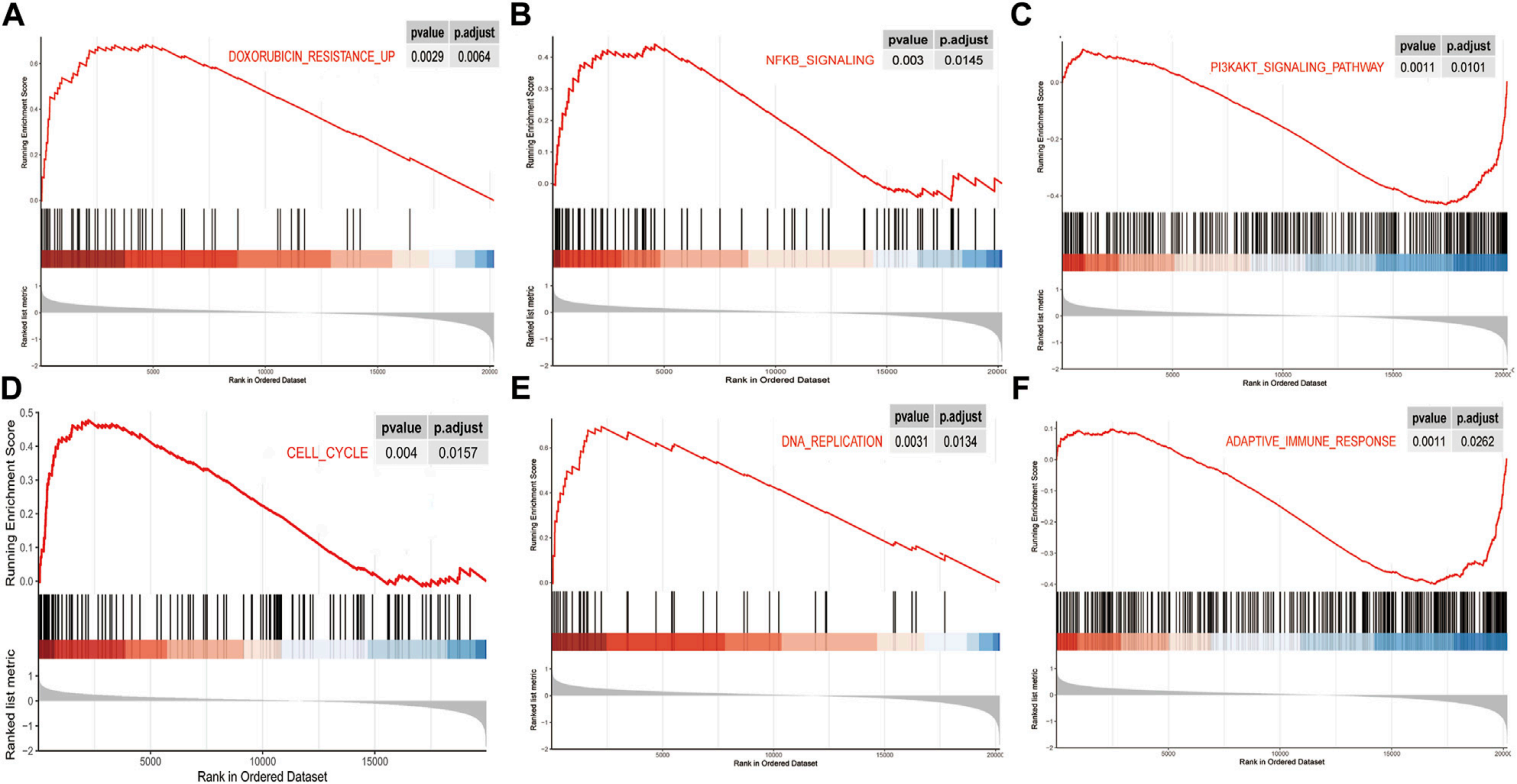
Pathway characteristics of gene signature in DLBCL. **(A-F)** Gene set enrichment analysis revealed pathways enriched in the high-risk group and low-risk group.

To investigate the potential functions and signaling pathways related to the gene signature in DLBCL, we used the five genes as baits to hook 501 highly relevant genes by the correlation test. GO analysis indicated that these genes were associated with cell adhesion, immune cell activation, and differentiation, as well as cytokine and growth factor-binding reaction (Figure 8A). KEGG analysis indicated that the 501 genes were involved in protein digestion and absorption, focal adhesion, and ECM-receptor interaction (Figure 8B). In addition, the differentially expressed genes between low-risk and high-risk groups were mainly enriched in the PI3K-AKT signaling pathway, focal adhesion, protein digestion and absorption, ECM-receptor interaction, and other pathways (Supplementary Figure S3). In the GSEA analysis between low-risk and high-risk groups, we found that the high-risk group was significantly associated with doxorubicin resistance (NES = 2.507 and p.adjust = 0.0064), NF-κB pathway (NES = 1.726 and p.adjust = 0.0145), cell cycle (NES = 2.052 and p.adjust = 0.0157), and DNA replication pathway (NES = 2.446 and p.adjust = 0.0134)(Figures 9A,B,D,E), while the low-risk group was associated with the activation of the PI3K-AKT pathway (NES = −1.793 and p.adjust = 0.0101) and adaptive immune response (NES = −1.683 and p.adjust = 0.0262) (Figures 9C,F).

### Infiltration Characteristics of TME Cells and Immune Response in the High-Risk and Low-Risk Groups

**FIGURE 10:**
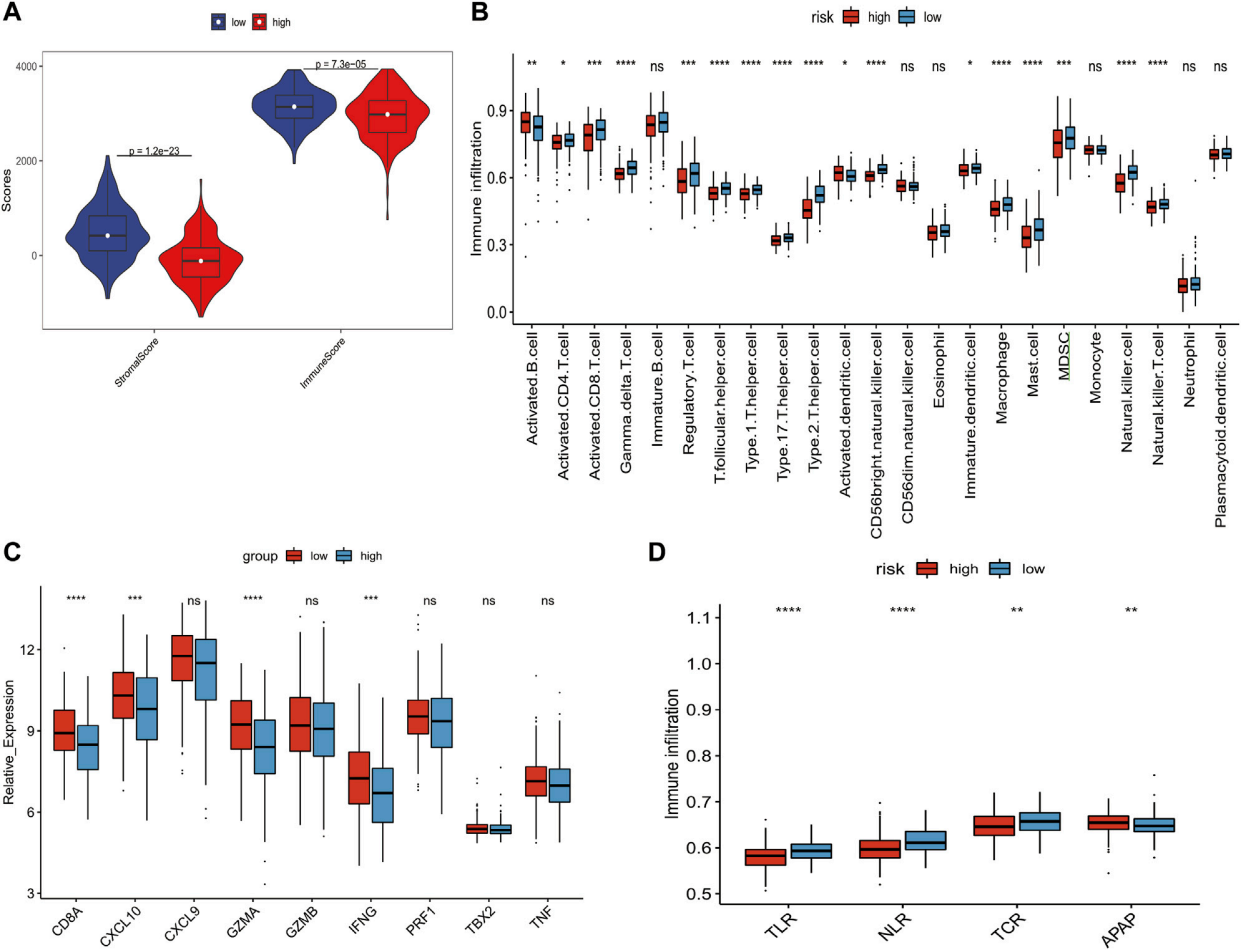
Correlation between gene signature and the tumor microenvironment and immune reaction. **(A)** Differences of immunescore and stromalscore (calculated by the ESTIMATE algorithm) between high-risk and low-risk patients. **(B)** Abundance of each TME cell in high-risk and low-risk groups. **(C)** Difference in the immune-activation–related gene expression between high-risk and low-risk patients. **(D)** Differences in immune-activated pathways between high-risk and low-risk patients. TLR, Toll-like receptor; NLR, NOD-like receptor; TCR, T-cell receptor; APAR, antigen processing and presentation. The statistical difference of two groups was compared by the Wilcoxon test. *p < 0.05; **p < 0.01; ***p < 0.001; and ****p < 0.0001.

We used the ESTIMATE algorithm to quantify the overall infiltration of immune and stromal cells between high-risk and low-risk patients. The results obtained indicated that there was significant higher immune and stroma cell infiltration in the low-risk patients (Figure 10A). To better elaborate the aforementioned findings, subsequent analysis of ssGSEA indicated that the low-risk group was rich in not only innate immune cell infiltration (e.g., dendritic cells, myeloid-derived suppressor cells, macrophages, mast cells, and natural killer cells) but also adaptive immune cell infiltration (e.g., activated CD4 T cells, activated CD8 T cells, gamma delta T cells, regulatory T cells, T follicular helper cells, type 1 T helper cells, type 17 T helper cells, and type 2 T helper cells) (Figure 10B). As expected, the low-risk group showed higher expression of mRNA related to immune activation and more obvious activation of immune pathways, including antigen processing and presentation pathways, NOD-like receptors, T-cell receptors, and Toll-like receptor pathways (Figures 10C,D). In summary, there was a higher degree of immune cell infiltration and immune activation response in the low-risk group.

### Difference in Tumor Stemness Between High-Risk and Low-Risk Groups

**FIGURE 11:**
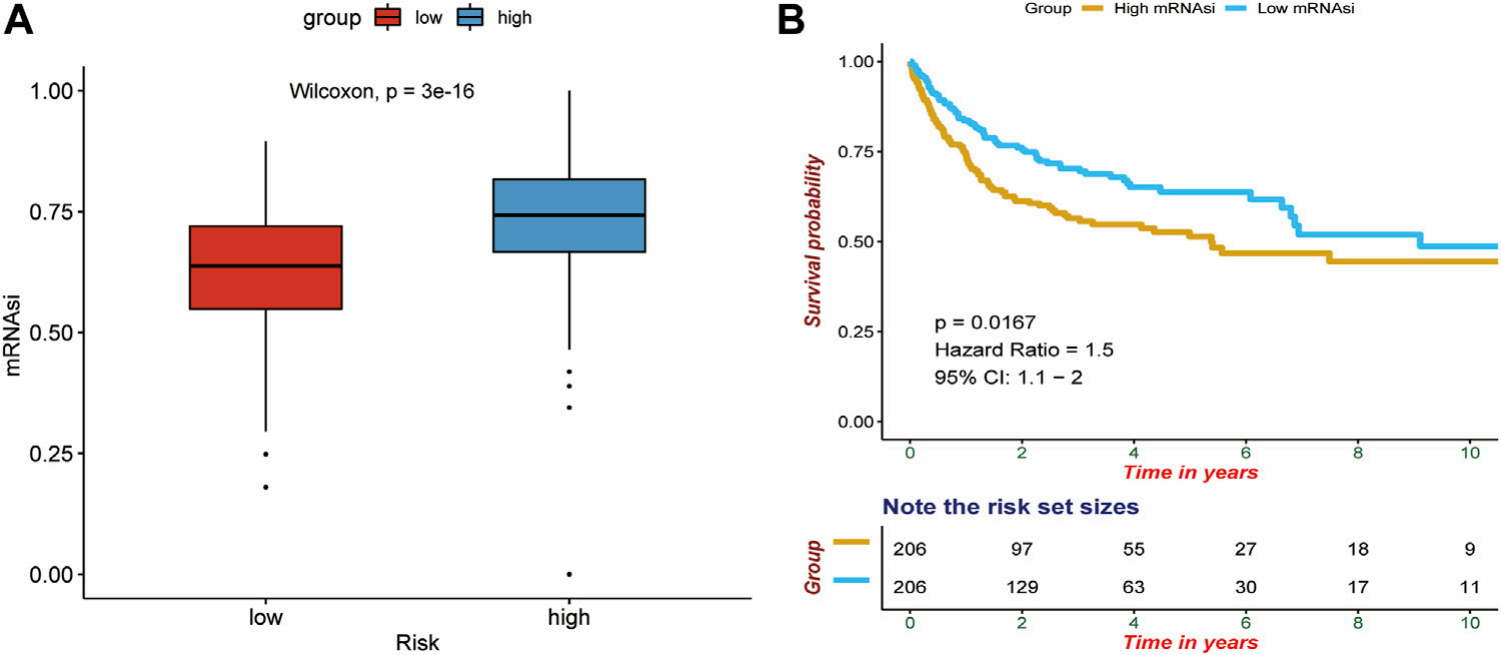
Correlation between the gene signature and tumor stemness. **(A)** Differences of mRNAsi between high-risk and low-risk patients. **(B)** Survival difference between high-mRNAsi and low-mRNAsi. *p*-values were calculated with the log-rank test.

We implemented the OCLR algorithm to obtain the mRNAsi of DLBCL. As shown in Figure 11A, patients in the high-risk group were more likely to have higher mRNAsi, suggesting that patients in the high-risk group had higher similarity to cancer stem cells, presenting more active biological processes and higher tumor dedifferentiation degree. In addition, the high-mRNAsi group showed a worse prognosis than the low-mRNAsi group (Figure 11B).

## Discussion

Diffuse large B-cell lymphoma is a highly heterogeneous tumor with different biological and clinical characteristics, so there is a large difference in the survival rate between the high-risk and low-risk patients (Juskevicius et al., 2016). Due to several limits in the present prognostic assessment system, it is urgent to establish a new model containing genetic information to better predict the prognosis of DLBCL patients (Ruppert et al., 2020).

The study of autophagy is a rapidly evolving field with great potential for providing new horizons for the treatment of malignant disease. Autophagy can regulate the components of the immune system, thereby affecting its homeostasis, survival, activation, proliferation, and differentiation (Jiang et al., 2019). At the same time, autophagy is also shown to enhance chemoresistance and helps in maintaining the stemness of tumor stem cells (Jang et al., 2017). Xu et al. (2021)uncovered that ARRDC1-AS1 facilitated the progression of DLBCL and enhanced autophagy of DLBCL by targeting the miR-2355-5p/ATG5 axis. Additionally, Amaravadi et al. (2007)found that inhibition of autophagy with either chloroquine or ATG5 short hairpin RNA (shRNA) enhanced the ability of either p53 activation or alkylating drug therapy to induce lymphoma cell death.

In this study, we successively used univariate Cox regression analysis, LASSO Cox regression analysis, and multivariate Cox regression analysis to screen autophagy-related prognostic genes and finally identified five genes to construct gene signature. However, unlike other research, we used two sources of autophagy-related genes from Hadb and GeneCards databases, respectively, to avoid missing some important genes that were fundamental to the prognosis of DLBCL. Meanwhile, to incorporate into genes with higher relevance to autophagy, we defined an association score higher than 7 as autophagy-related genes. Our results showed that this gene signature could effectively classify patients into high-risk and low-risk groups with significant differences in overall survival and progression-free survival and had favorable prognostic accuracy. The nomogram consisted of the gene signature and clinical factors had better discrimination and prognostic stratification ability than the gene signature and IPI score alone, and it also had a favorable consistency between the predicted and actual survival. To the best of our knowledge, the gene prognostic models have been acknowledged by the majority of researchers, but there were few studies concerning DLBCL. Pan et al. constructed a TME-relevant gene signature that could not only predict prognosis but also explored the relationship between the TME and DLBCL (Pan et al., 2021). The autophagy-related gene signature outlined in our study was the first explanation toward the pathophysiological process of diffuse large B-cell lymphoma with poor prognosis from the perspective of autophagy.

The results of GSEA analysis revealed the differences in pathways that might lead to different prognosis in high- and low-risk groups. Doxorubicin resistance, NF-κB pathway, cell cycle, and DNA replication pathway were activated in high-risk patients. The PI3K-AKT signaling pathway and adaptive immune response pathway were activated in low-risk patients. It is known that the abnormal regulation of the cell cycle and DNA replication played a crucial role in promoting tumor growth (Song et al., 2021). The previous study showed that constitutive activation of NF-κB was characteristic of most ABC subtypes of DLBCL, and the activation of the NF-κB pathway may be one of the mechanisms resulting in drug resistance of relapsed/refractory DLBCL (Turturro, 2015). For the PI3K-AKT signaling pathway enriched in the low-risk group, it could inhibit the activation of autophagy (M. Martelli et al., 2011). There was a good consistency with the findings of recent studies. Xie and his colleagues constructed an RNA binding protein-based prognostic signature for diffuse large B-cell lymphoma and also found that the activation of autophagy was associated with high-risk patients who had poor outcomes (Xie et al., 2021).

In addition, we also found that the low-risk group exhibited higher immune cell infiltration. The latest research showed that targeting autophagy pathways could reshape the tumor microenvironment by improving antigen processing and presentation to enhance T-cell response or increasing the production of Th1 chemokines to promote the infiltration of effector immune cells (Xia et al., 2021). A phase II clinical study also showed that after hydroxychloroquine was used in combination with the chemotherapy drugs paclitaxel and gemcitabine, patients with pancreatic adenocarcinoma showed increased immune cell infiltration (Zeh et al., 2020). Moreover, there was a higher degree of autophagy in the high-risk group, and patients in the high-risk group might have higher mRNAsi, indicating that these patients were more likely to have characteristics such as chemotherapy resistance and more invasiveness like CSCs. Therefore, the application of autophagy inhibitors might be a potentially important strategy for anti-tumor therapy.

The five genes identified in our study have previously been correlated with the prognosis of tumors. PRKCQ is a member of the novel protein kinase C (PKC) family and has been associated with many types of cancers, such as chromophobe renal cell carcinomas, breast cancer, and Notch-driven T-cell leukemia (Villalba and Altman, 2002; Byerly et al., 2016; Park et al., 2017). According to the GeneCards database, tumor suppressor candidate gene 1 (TUSC1) is located on chromosome 9p and is downregulated in non–small cell lung cancer and small cell lung cancer cell lines, suggesting that it may play a role in lung tumorigenesis. Tumor Protein P53 Inducible Nuclear Protein 2 (TP53INP2), which has about 36% homology with the known tumor protein 53-induced nuclear protein 1, plays a role in carrying LC3 and its homologous proteins out of the nucleus to autophagosome and promoting the interaction of LC3 and its homologous proteins with ATG7 (Liu and Klionsky, 2015). Current research shows that TP53INP2 either can promote the development or inhibit the proliferation of tumor cells depending on the tumor types (He et al., 2015; Hu et al., 2017). AMPK is a heterotrimer consisting of an alpha catalytic subunit and non-catalytic beta and gamma subunits. Among them, the beta1 subunit was encoded by PRKAB1. A finding indicated that the overexpression of AMPK-β1 inhibited the proliferation, migration, and invasion of ovarian cancer cells. When siRNA was used to interfere with AMPK-β1, the invasion ability of tumor cells would be enhanced (Li et al., 2014). As a protein-coding gene, HIF-1A encoded the alpha subunit of transcription factor hypoxia-inducible factor-1 (HIF-1). Overexpression of HIF-1α has been reported in several solid tumors, and elevated HIF-1α protein levels correlate with poor prognosis in majority of tumors (Zhong et al., 1999). However, Evens et al. (2010)revealed that expression of HIF-1α was an independent favorable prognostic factor affecting the overall survival in DLBCL patients receiving RCHOP regimen. Nevertheless, despite the proposed functions of these five ARGs in various types of cancer, the specific role of these genes in patients with DLBCL remains unclear.

Inevitably, there were still some potential limitations that could not be neglected in the present study. First, there are unknown biases because of the retrospective nature of our data. Second, the information of several clinical data of some patients was unavailable in these public datasets, and these patients need to be excluded in some analysis. Third, there were only autophagy-related genes included in our study, which did not represent the entire gene transcription profile associated with DLBCL. Fourth, there was a lack of further experiments to identify the function of these genes. Hence, the value of this gene signature is preliminarily demonstrated, and further verification is expected.

## Conclusion

Taken together, we identified an autophagy-related gene signature that could efficiently predict the overall survival of DLBCL patients and was independent of other clinical factors. Moreover, the gene signature might serve as a promising marker of therapeutic resistance in DLBCL patients.

## Data Availability

Publicly available datasets were analyzed in this study. These data can be found at: https://cancergenome.nih.gov/ https://www.ncbi.nlm.nih.gov/gds/?term=.
