# Supplementary figures and images for: An Autophagy-Related Gene Signature can Better Predict Prognosis and Resistance in Diffuse Large B-Cell Lymphoma
Source: Front Genet. 2022 Jun 30;13:862179. doi: 10.3389/fgene.2022.862179 (PMC9280409; doi:10.3389/fgene.2022.862179)

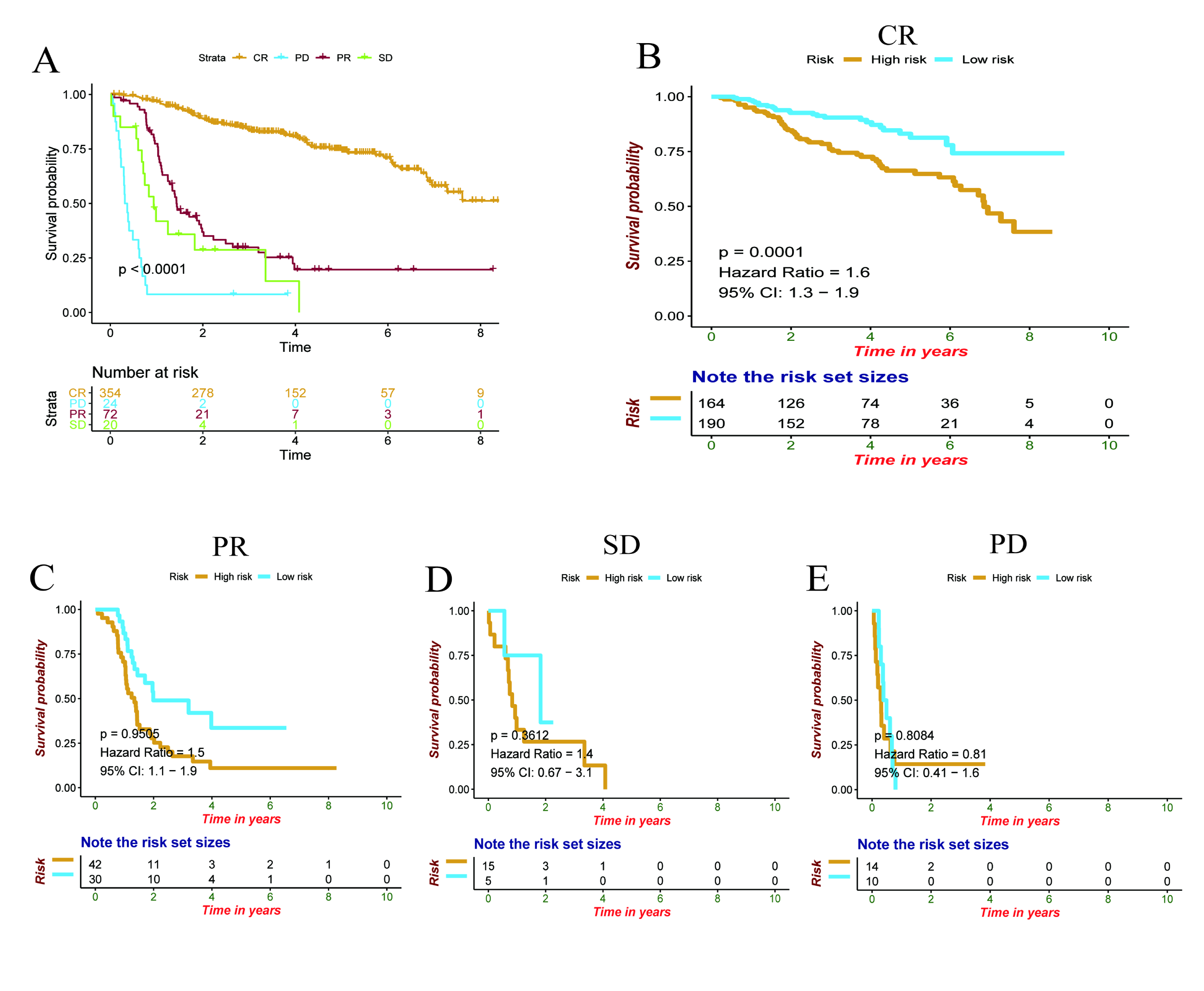

Supplement: Supplementary file 1 [file Image2.TIF]

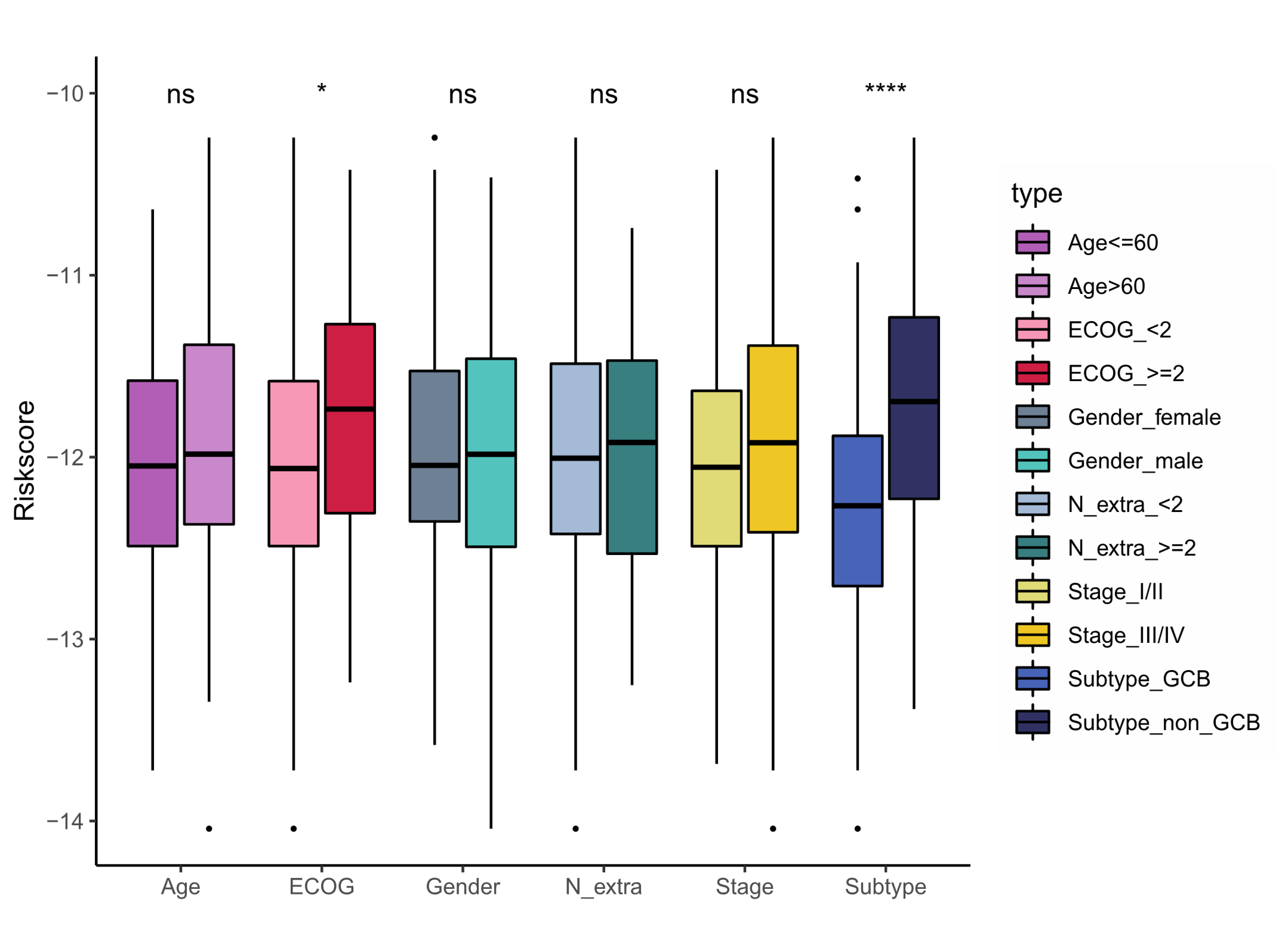

Supplement: Supplementary file 2 [file Image1.TIF]

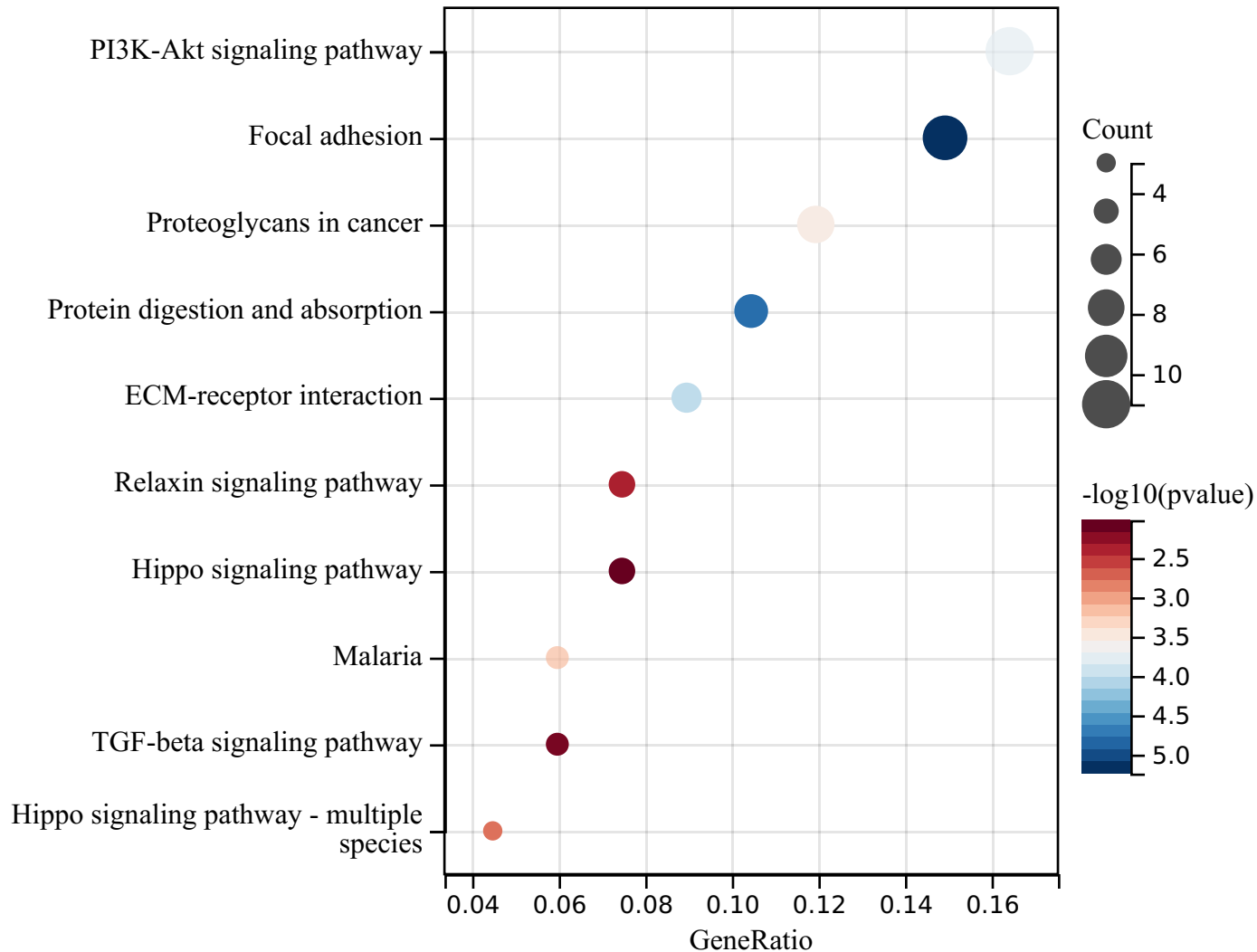

Supplement: Supplementary file 3 [file Image3.pdf]
