# Supplementary material for: An Autophagy-Related Gene Signature can Better Predict Prognosis and Resistance in Diffuse Large B-Cell Lymphoma
Source: Front Genet. 2022 Jun 30;13:862179. doi: 10.3389/fgene.2022.862179 (PMC9280409; doi:10.3389/fgene.2022.862179)
Supplement: Supplementary file 4 [file DataSheet1.docx]

**Figure S1.** Correlation between gene signature and clinical parameters.

**Figure S2.** The ability of gene signature to distinguish the prognosis of patients with different treatment responses. (A) Patients with different treatment responses had different prognosis. (B) Patients with the complete response state could be divided into two groups with significant difference of overall survival by gene signature. (C-E) Patients with a partial response, stable disease or progressive disease could not be divided into two groups with significant difference of overall survival by gene signature.

**Figure S3.** Gene functional enrichment of differentially expressed genes between high-risk and low-risk groups**.**
